# Gum Arabic as a potential candidate in quorum quenching and treatment of periodontal diseases

**DOI:** 10.3389/froh.2024.1459254

**Published:** 2024-10-08

**Authors:** Nada Tawfig Hashim, Rasha Babiker, Mohammed Mustahsen Rahman, Nallan C. S. K. Chaitanya, Riham Mohammed, Shahistha Parveen Dasnadi, Bakri Gobara Gismalla

**Affiliations:** ^1^ Department of Periodontics, RAK College of Dental Sciences, RAK Medical & Health Sciences University, Ras al-Khaimah, United Arab Emirates; ^2^ Department of Physiology, RAK College of Medical Sciences, RAK Medical & Health Sciences University, Ras-al-Khaimah, United Arab Emirates; ^3^ Department of Oral Radiology, RAK College of Dental Sciences, RAK Medical & Health Sciences University, Ras al-Khaimah, United Arab Emirates; ^4^ Department of Oral Surgery, RAK College of Dental Sciences, RAK Medical & Health Sciences University, Ras al-Khaimah, United Arab Emirates; ^5^ Department of Orthodontics, RAK College of Dental Sciences, RAK Medical & Health Sciences University, Ras al-Khaimah, United Arab Emirates; ^6^ Department of Oral Rehabilitation, Faculty of Dentistry, University of Khartoum, Khartoum, Sudan

**Keywords:** Gum Arabic (GA), periodontal disease, biofilm, quorum sensing, quorum quenching, antibacterial, anti-biofilm, anti-inflammatory

## Abstract

Periodontal diseases are chronic inflammatory conditions influenced by bacterial biofilm formation and host immune responses, affecting millions worldwide. Traditional treatments like mechanical debridement and systemic antibiotics often face limitations, including biofilm resilience and antibiotic resistance. Gum Arabic (GA), a natural exudate from *Acacia* trees, presents a promising alternative with its anti-biofilm and anti-inflammatory properties. This review highlights the role of GA in periodontal therapy, particularly its ability to interfere with quorum sensing (QS) pathways, specifically the AI-2 signaling system used by key periodontal pathogens such as *Porphyromonas gingivalis*, *Aggregatibacter actinomycetemcomitans*, and *Fusobacterium nucleatum*. By disrupting QS, GA inhibits biofilm formation, reduces bacterial virulence, and promotes a balanced oral microbiome. GA's prebiotic properties also encourage the growth of beneficial bacteria, enhancing the host's immune response while preserving the systemic microbiome. Clinical studies demonstrate GA's effectiveness as an adjunct in periodontal therapy, with significant reductions in plaque accumulation, gingival inflammation, and bleeding. This highlights GA's potential as a natural therapeutic agent, offering an effective, antibiotic-sparing option in managing periodontal disease. However, further research is warranted to fully establish GA's role in comprehensive periodontal care and its long-term benefits.

## Introduction

Periodontal diseases are complex, chronic inflammatory conditions influenced by biofilm formation and the host immune response (1).

They are among the most prevalent diseases worldwide, affecting up to 50% of the adult population, with severe periodontitis being the 11th most common disease globally (2).

Periodontitis can lead to tooth loss, negatively impacting aesthetics, nutrition, and overall quality of life. Moreover, it imposes significant economic burdens on healthcare systems due to the costs associated with treatment and management (3–6).

Periodontitis is a chronic inflammatory condition that results from an imbalance between the host immune response and bacterial pathogens such as *Porphyromonas gingivalis*, *Treponema denticola*, *Tannerella forsythia*, and *Aggregatibacter actinomycetemcomitans* (7). These pathogens produce virulence factors, including collagenases, sulfides, and endotoxins, which contribute to tissue damage. Various factors, such as dry mouth, biofilm retention, smoking, metabolic conditions, poor nutrition, certain medications, systemic disorders, and stress, can exacerbate this dysbiotic microbial environment.

**Figure 1:**
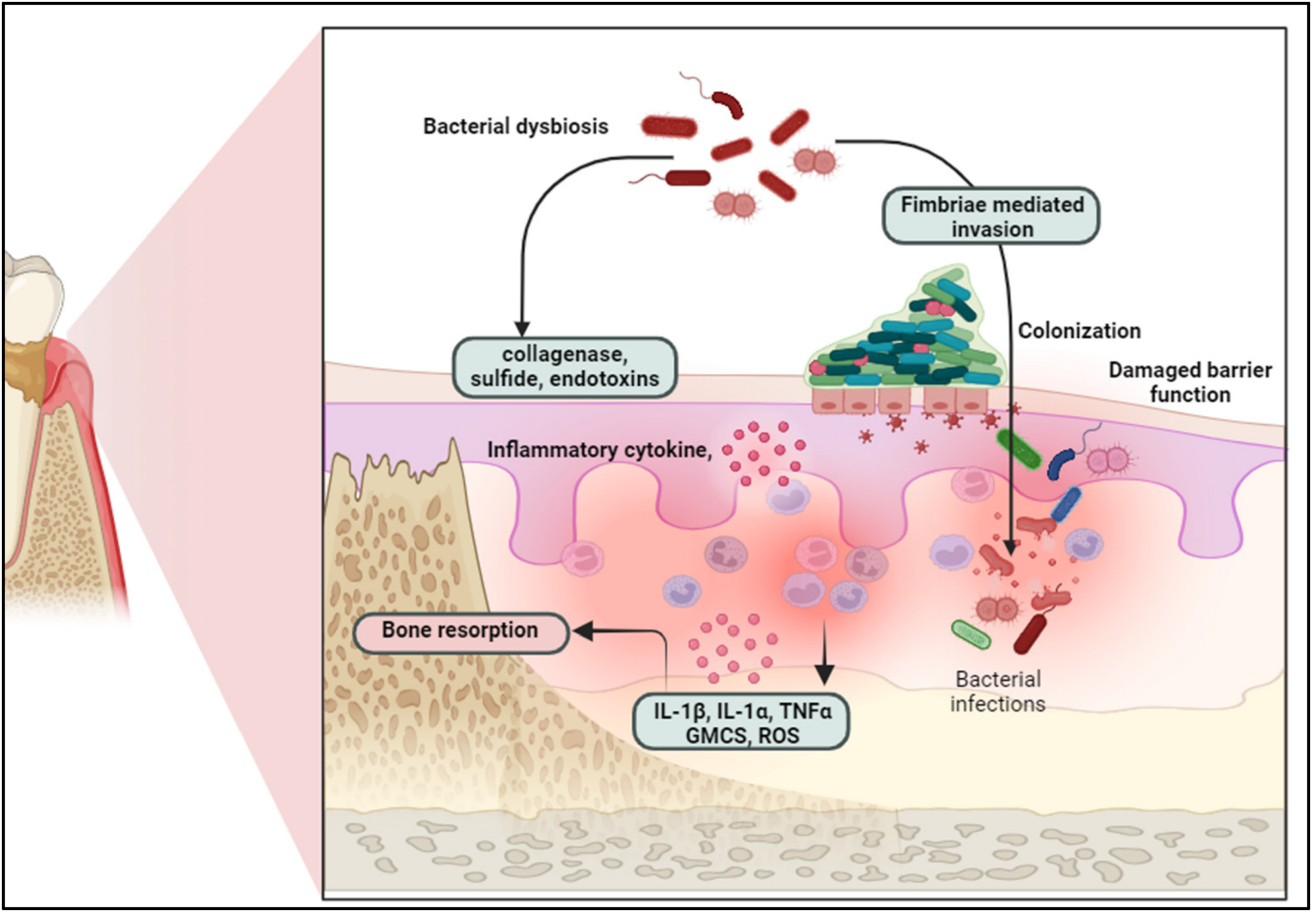
Bacterial dysbiosis triggers epithelial barrier disruption, bacterial colonization, and immune response, leading to inflammatory cytokine release and bone resorption in periodontal disease progression. Created with BioRender.com.

Dysbiotic microbiome may trigger an immune response, leading to the production of pro-inflammatory cytokines such as IL-1β, IL-6, IL-1α, TNF-α, and GM-CSF. If this trigger is persistent it may result in a chronic inflammatory state that leads to the irreversible destruction of connective tissue and alveolar bone, driving the initiation and progression of periodontitis (8, 9) (Figure 1). The importance of treating periodontal disease extends beyond oral health. Periodontal infections have been linked to systemic conditions such as cardiovascular disease, diabetes, respiratory diseases, and adverse pregnancy outcomes (10–12).

The systemic inflammation induced by periodontal pathogens and their toxins can exacerbate these conditions, highlighting the need for effective periodontal therapy.

Traditional treatments for periodontal disease often include mechanical debridement (scaling and root planing) and the use of systemic or local antibiotics (13). However, these approaches face limitations due to the resilience of biofilms and the growing concern of antibiotic resistance. Biofilms protect bacteria from external threats, making them difficult to eradicate with standard treatments (14, 15). This has spurred interest in alternative therapeutic strategies that can effectively disrupt biofilms and modulate the host immune response without contributing to antibiotic resistance.

Gum Arabic (GA), a natural exudate from *Acacia* trees, presents a novel approach with the potential for anti-biofilm and anti-inflammatory properties (16). Composed primarily of polysaccharides and proteoglycans, GA has been traditionally used for its medicinal properties, including its ability to soothe inflammation and promote wound healing (17).

Recent research suggests that GA may also have specific actions against biofilm formation and quorum sensing (QS) in periodontal pathogens, offering a promising adjunct to conventional periodontal therapy (18).

## Formation of biofilms via quorum sensing

Biofilms are intricate microbial communities embedded in a self-produced extracellular matrix (19, 20). They play a pivotal role in periodontal diseases by enabling bacterial persistence and resistance to treatment (19).

The bacteria within biofilms communicate via quorum sensing (QS), a cell-to-cell signaling mechanism that coordinates collective behaviors beneficial to the bacterial community (21).

Quorum sensing enables bacteria in biofilms to chemically communicate and coordinate their activity as if they were multicellular organisms in response to environmental changes. Bacteria produce and export signaling molecules known as autoinducers (AIs), which are discharged into the environment and built up at a concentration corresponding to cell density, which is the basis of the classical QS signaling system (22).

**Figure 2:**
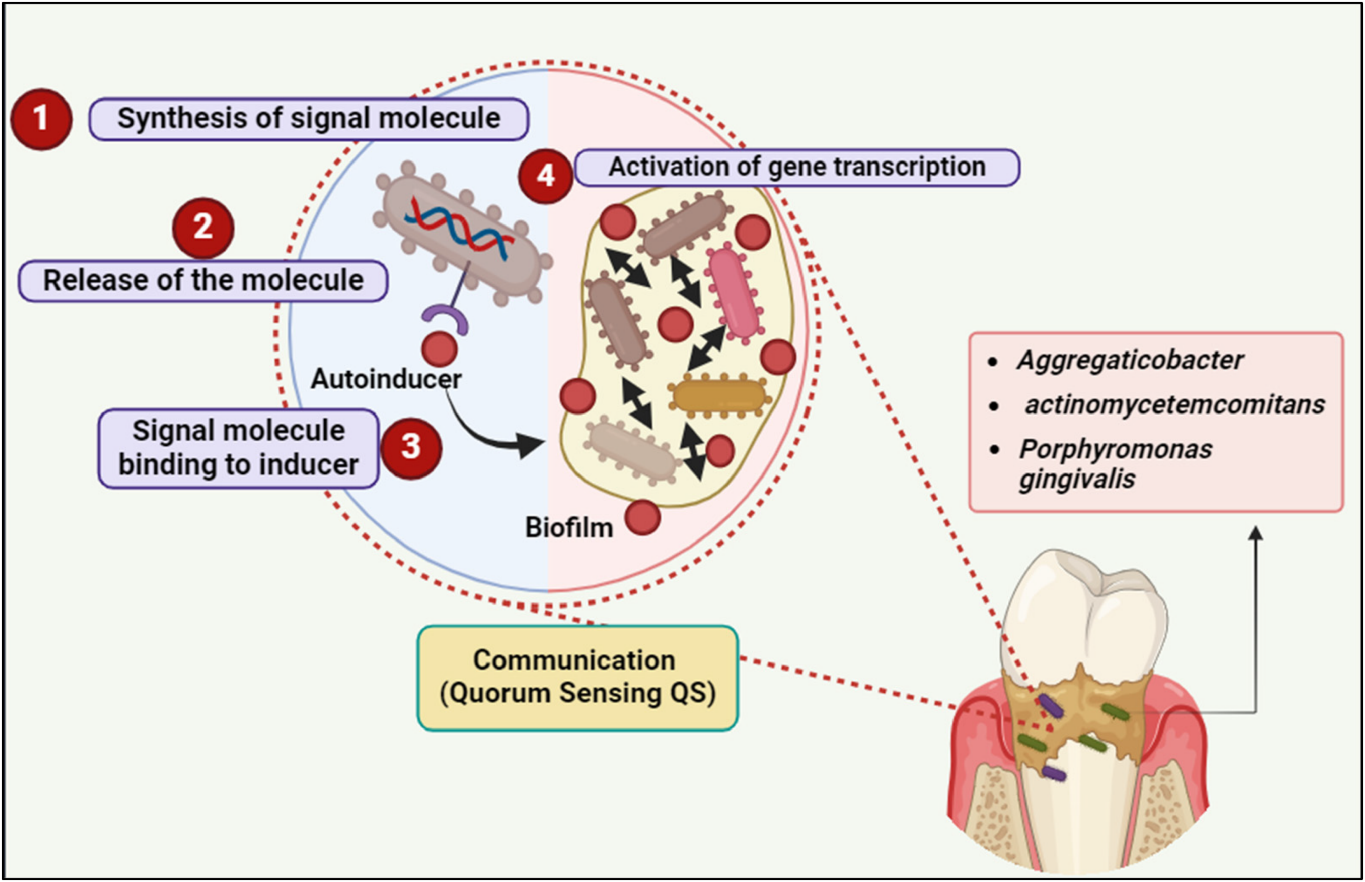
Quorum sensing (QS) communication in biofilm formation involves four key steps: signal molecule synthesis, release, binding to the inducer, and activation of gene transcription, facilitating bacterial communication and biofilm development by species such as *Aggregatibacter actinomycetemcomitans* and *Porphyromonas gingivalis*. Created with BioRender.com.

The autoinducer can attach to and activate a corresponding receptor protein, which in turn triggers changes in gene expression, when a certain concentration of this signal is obtained and, consequently, a “quorum” of cells is present in a specific environment (23, 24) (Figure 2).

Bacteria use a variety of autoinducers; for example, Gram-negative bacteria frequently emit N-acyl L-homoserine lactone (AHL) derivatives, Gram-positive bacteria release peptide signals, and many species in both categories detect the interspecies signal AI-2. QS pathways regulate a diverse spectrum of gene products and phenotypes, including bioluminescence in marine bacteria and the generation of toxins and virulence factors by specific microbes (25).

When a threshold concentration of autoinducers is reached, these molecules trigger changes in gene expression, leading to coordinated activities like biofilm formation and virulence factor production (23, 24).

**Figure 3:**
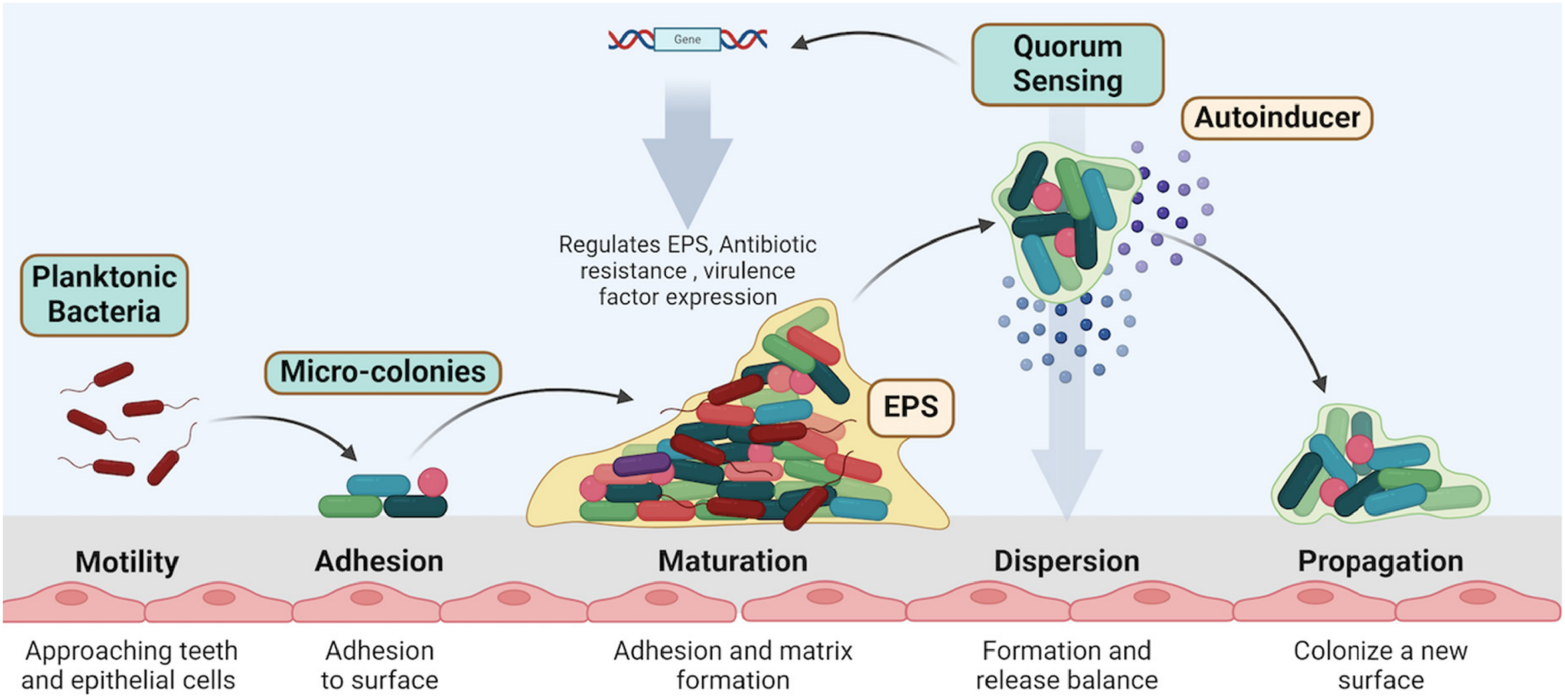
Biofilm formation involves key stages: motility and adhesion of planktonic bacteria, followed by micro-colony maturation, matrix formation, and quorum sensing regulation. Dispersed bacteria propagate to new surfaces, continuing the biofilm lifecycle. Created with BioRender.com.

The formation of biofilms begins with the initial adhesion of planktonic (free-floating) bacteria to a surface (26, 27) (Figure 3). This process is facilitated by bacterial appendages like fimbriae and pili, which allow bacteria to adhere to surfaces in the oral cavity, such as teeth and epithelial cells. Once attached, the bacteria begin to multiply and form microcolonies (28). During this stage, the bacteria produce extracellular polymeric substances (EPS), a matrix composed of polysaccharides, proteins, and nucleic acids. This matrix protects the bacteria from environmental stressors and facilitates further adhesion of other microbial species (29).

As the biofilm matures, the microcolonies expand and merge, forming a complex, three-dimensional structure. This structure is highly organized, with channels that allow for nutrient and waste exchange (28, 30) (Figure 3). Quorum sensing plays a critical role at this stage by regulating the production of EPS and other factors necessary for biofilm integrity (31).

Bacteria within the biofilm produce and release signaling molecules known as autoinducers. The concentration of these molecules increases as the bacterial population grows. When the concentration of autoinducers reaches a threshold, bacteria detect these signals through specific receptors (32). This detection triggers a coordinated response, altering gene expression to promote biofilm maturation and maintenance (32).

Quorum sensing regulates various genes involved in biofilm formation, including those responsible for EPS production, antibiotic resistance, and virulence factor expression. For example, Gram-negative bacteria commonly use N-acyl homoserine lactones (AHLs) as autoinducers, while Gram-positive bacteria use oligopeptides (33).

In the final stage of the biofilm life cycle, some bacteria may disperse from the biofilm to colonize new surfaces. This dispersal can be triggered by changes in environmental conditions or nutrient availability. Dispersal is also regulated by quorum sensing, ensuring that bacteria can respond adaptively to their environment (34, 35) (Figure 3).

## Effect of quorum sensing on the virulence of the periodontal pathogens

Quorum sensing (QS) not only regulates biofilm formation but also significantly influences the virulence of periodontal pathogens (36). The ability of bacteria to communicate and coordinate their activities enhances their pathogenicity and adaptability within the host environment (36).

QS systems control the expression of genes encoding virulence factors, such as proteases, toxins, and adhesins. In periodontal pathogens like *Porphyromonas gingivalis* and *Aggregatibacter actinomycetemcomitans*, QS regulates the production of these factors, contributing to tissue destruction and disease progression (37, 38).

By coordinating the production of virulence factors, QS enhances the ability of bacteria to evade host immune responses (36). For example, QS-regulated proteases can degrade host immune molecules, while other factors can modulate the host inflammatory response to create a more favorable environment for bacterial survival. QS is crucial for maintaining the stability and integrity of the biofilm matrix (39). The production of extracellular polymeric substances (EPS) and other matrix components is tightly regulated by QS, ensuring the biofilm's resilience against physical and chemical challenges, including host immune attacks and antimicrobial treatments (40).

QS also facilitates communication not only within a single bacterial species but also between different species within the biofilm (41). This interspecies communication can enhance the overall virulence of the microbial community (42). For instance, *Fusobacterium nucleatum* acts as a bridge organism, linking early colonizers with late colonizers like *P. gingivalis*, thereby promoting a pathogenic biofilm community (43). Furthermore, QS enables bacteria to rapidly adapt to changes in their environment, such as nutrient availability or the presence of antimicrobial agents. This adaptability is critical for the persistence of periodontal pathogens in the dynamic environment of the oral cavity (44).

A key factor in the development of periodontal disease is quorum sensing. Most quorum-sensing research on periodontopathic biofilms is based on Fusobacterium nucleatum and the red complex pathogens (45). Periodontal diseases have a huge worldwide burden and have been linked to several conditions. It is therefore not surprising that research has looked for alternate antibacterial strategies in recent years (3–5).

Dental plaque is a key contributing factor to periodontal disease, hence scaling and root planing are used as a mechanical means of removing plaque (46). Therapeutic plaque control medications are frequently regarded as adjuvant therapies when this is not adequate. Nevertheless, these treatments frequently have unfavorable side effects including tooth discoloration and unpleasant tastes (47, 48). Maintaining the normal oral flora while carefully adjusting the host immune response are the goals of new treatments for periodontal diseases (49).

New findings in quorum quenching, or quorum sensing suppression, point to a possible antibacterial strategy to reduce the toxicity of oral biofilms by blocking bacterial communication networks. Quorum quenchers can be manufactured synthetically or extracted naturally from bacteria, plants, or human saliva (45, 50).

## Quorum quenching: a novel approach

It would appear that naturally existing quorum-quenching mechanisms play important roles in the interactions between microbe-microbe and pathogen-host interactions. The discovery of enzymes that degrade quorum-sensing signals in mammalian species represents a significant step forward in the field of research on quorum sensing and quenching (51).

One potential strategy for interfering with the formation of the plaque biofilm is to regulate the quorum-sensing mechanism utilized by various bacterial species. Quorum quenching, often known as QQ, is a term widely used to refer to the interference of quorum sensing (52).

Quorum quenching (QQ) involves disrupting QS pathways, thereby inhibiting biofilm formation and bacterial virulence. This strategy offers a promising alternative to conventional antibiotics, which often lead to resistance (53).

Natural and synthetic compounds can serve as quorum quenchers, providing an innovative approach to managing biofilm-associated infections like periodontal disease (54). Many of these chemicals are not antibiotics, and bacteria do not quickly develop resistance to them. After that, the antibiofilm impact is typically long-lasting. Numerous uses in the food, pharmaceutical, and other sectors are made possible because these substances are often harmless to eukaryotic organisms. Sometimes the ultimate impact of these substances is understood, while in other instances their precise mechanism of action in preventing the formation of biofilms is known (54).

## Efficacy of Gum Arabic in oral health

Medicinal plants and their derivatives have long been regarded as safe medicinal agents for use in humans and as a key component of the pharmaceutical industry (55).

Gum secretions are released by leguminous and acacia trees, which are found around the world. These secretions have long been utilized as an anti-inflammatory and in the treatment of many ailments. These plant secretions can function as antibiotics against a wide range of bacterial acids that cause infections in humans (56).

Gum Arabic (GA), also known as acacia gum, is an exudate produced by the Acacia Senegal and Acacia Seyal trees, which are members of the Leguminosae family (57).

GA has the structure of an arabinogalactan-protein complex. This complex consists of Arabic acid salts in magnesium, calcium, and potassium. The GA structure consists of 1-3-linked β-D-galactopyranosyl units, with branches of two to five β-D-galactopyranosyl residues linked together by 1,3-ether linkages and connected to the core β-D-galactopyranosyl chain by 1,6-linkages (58).

GA has Anti-Biofilm Activity by interfering with QS, particularly the AI-2 signaling system used by many periodontal pathogens. This disruption can prevent biofilm formation and reduce bacterial virulence (59).

GA also exhibits broad-spectrum antibacterial effects, targeting both planktonic bacteria and those within biofilms (60).

The prebiotic effects of GA promote the growth of beneficial gut bacteria, leading to the production of short-chain fatty acids like butyrate, which have anti-inflammatory properties (61).

GA has shown potential in reducing antibiotic resistance by acting as a natural adjunct to traditional antimicrobial therapies (60). Its prebiotic properties promote the growth of beneficial bacteria in the gut and oral microbiome, which can outcompete pathogenic strains that may develop resistance (61). Additionally, GA enhances the body's immune response, helping to control infections more efficiently and reducing the need for systemic antibiotics (60). By supporting a balanced microbiome and targeting localized infections, GA limits the overuse of antibiotics, which is a key driver of resistance development. In addition, GA offers significant benefits by preserving the systemic microbiome, particularly the gut microbiome, which is often disrupted by systemic antibiotic use (61).

Antibiotics delivered systemically can lead to gut dysbiosis, antibiotic-associated diarrhea, and the overgrowth of harmful bacteria, such as *Clostridium difficile* (62). By limiting the treatment to the periodontal pockets, GA can prohibit these complications and maintains the body's microbial balance. Furthermore, the highly localized delivery of GA allows for more concentrated and effective treatment at the site of infection without systemic side effects, improving both efficacy and patient comfort (63). With regard to periodontal therapy, GA compounds have a great deal of promise to support microbiome homeostasis, especially when considering precision medicine. GA may be customized to a person's microbiome and immune response as opposed to using a one-size-fits-all strategy. Its prebiotic qualities promote the development of advantageous microorganisms while preventing the overabundance of harmful bacteria (64).

This targeted modulation is consistent with precision medicine, which focuses on individualized therapy approaches that take into account the distinct features of the patient's microbiome, immune system, and genetic profiles (65). By supporting the host's immune response and encouraging a balanced microbial environment, GA contributes to long-term periodontal health. Its ability to work synergistically with the host's immune system could be tailored to address specific microbiome imbalances in different patients, making it a valuable tool in personalized periodontal therapies. In this way, GA compounds may not adhere to a one-size-fits-all approach but could instead be a versatile component in individualized treatment regimens, enhancing the host-microbiome interaction for optimal outcomes (66, 67).

### Molecular mechanisms involved in GA protective action in oral health

**Figure 4:**
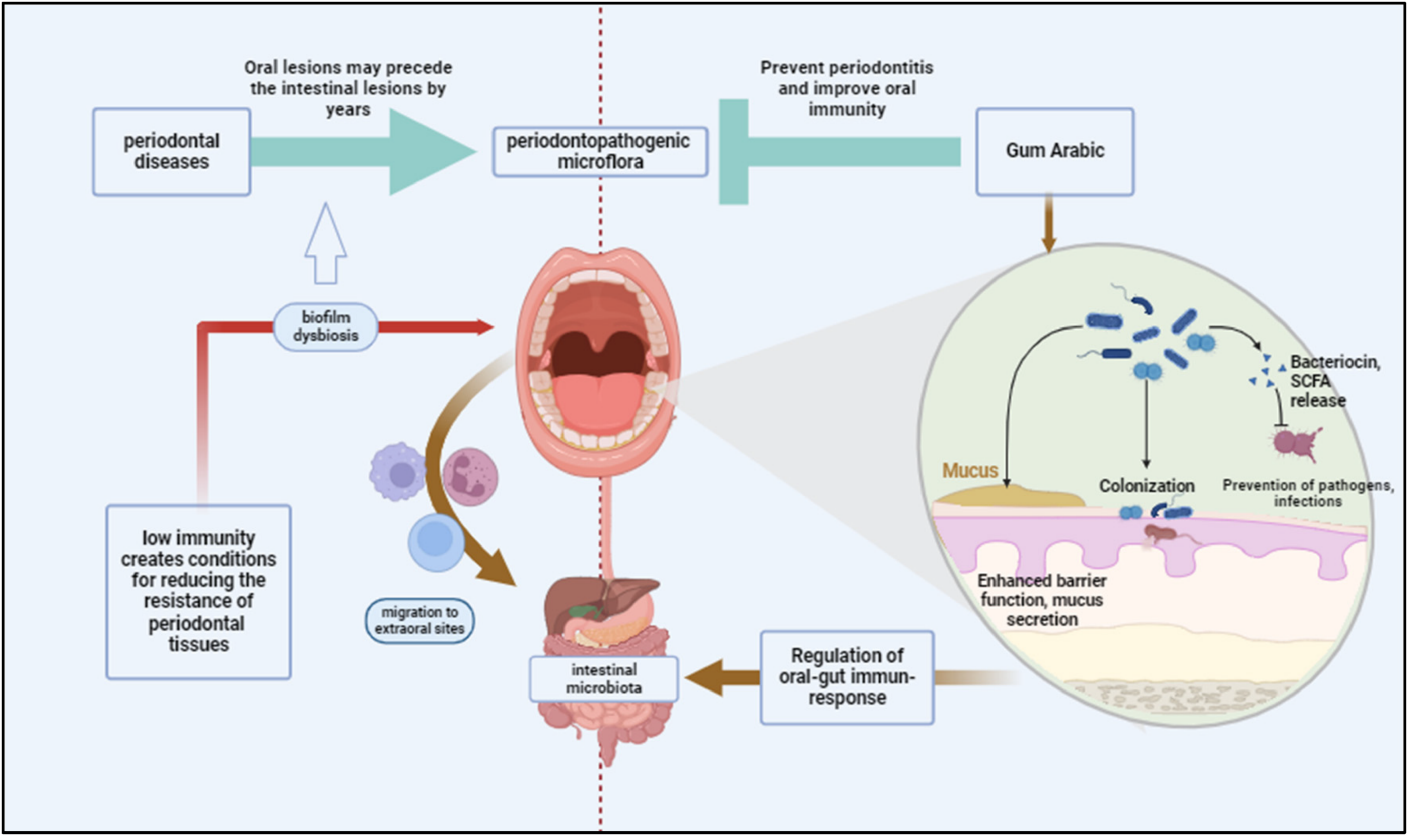
Periodontal diseases contribute to systemic dysbiosis by facilitating the migration of periodontopathogenic bacteria from the oral cavity to the intestines, potentially compromising immunity. Gum Arabic may prevent periodontitis by enhancing gut and oral immunity, improving barrier function, and promoting colonization by beneficial bacteria. Created with BioRender.com.

• **Interference with Autoinducer Production**: GA contains components like D-galactose, which can interfere with the production and function of autoinducers. D-galactose has been shown to inhibit the activity of AI-2, a common autoinducer in periodontal pathogens. This inhibition can disrupt the QS signaling necessary for biofilm formation and virulence factor production (60) (Figure 4).
• **Blocking Autoinducer Receptors**: GA can block the receptors that detect autoinducers, preventing the QS signals from being received and processed by the bacterial cells. This blockade hinders the bacteria's ability to coordinate their activities, thereby reducing biofilm formation and virulence (60).
• **Degradation of Signaling Molecules**: Some components of GA can degrade signaling molecules used in QS. By breaking down these molecules, GA disrupts the communication network within the biofilm, making it less stable and more susceptible to disruption (68).
• **Anti-Inflammatory Effects**: GA's anti-inflammatory properties are attributed to its ability to modulate the host immune response. Studies have demonstrated that GA can reduce the levels of pro-inflammatory cytokines, such as IL-6, and inhibit the activation of NFκB, a key regulator of inflammation (69). The prebiotic effects of GA also contribute to its anti-inflammatory properties by promoting the growth of beneficial gut bacteria that produce anti-inflammatory short-chain fatty acids like butyrate (70) (Figure 4).
• **Enhancing Host Defense Mechanisms**: By modulating the immune response and promoting the growth of beneficial bacteria, GA helps enhance the host’s natural defense mechanisms against periodontal pathogens. This dual action not only disrupts biofilm formation but also supports the resolution of inflammation and healing of periodontal tissues (71).
• **Induction of LL-37 Production**: Gum Arabic has been shown to influence the production of LL-37, primarily through the generation of butyrate, a short-chain fatty acid produced by the fermentation of dietary fibers by colonic bacteria (72). When GA, is fermented by gut microbiota, it produces butyrate. This compound has been demonstrated to upregulate the expression of LL-37 in human cells, thereby enhancing the innate immune response in the oral cavity. Butyrate induces the expression of the cathelicidin antimicrobial peptide (CAMP) gene, which encodes for LL-37. This process enhances the antimicrobial defense mechanisms of oral tissues (60, 72).

## Bioactive components of Gum Arabic and their potential role in quorum sensing and biofilm

**Figure 5:**
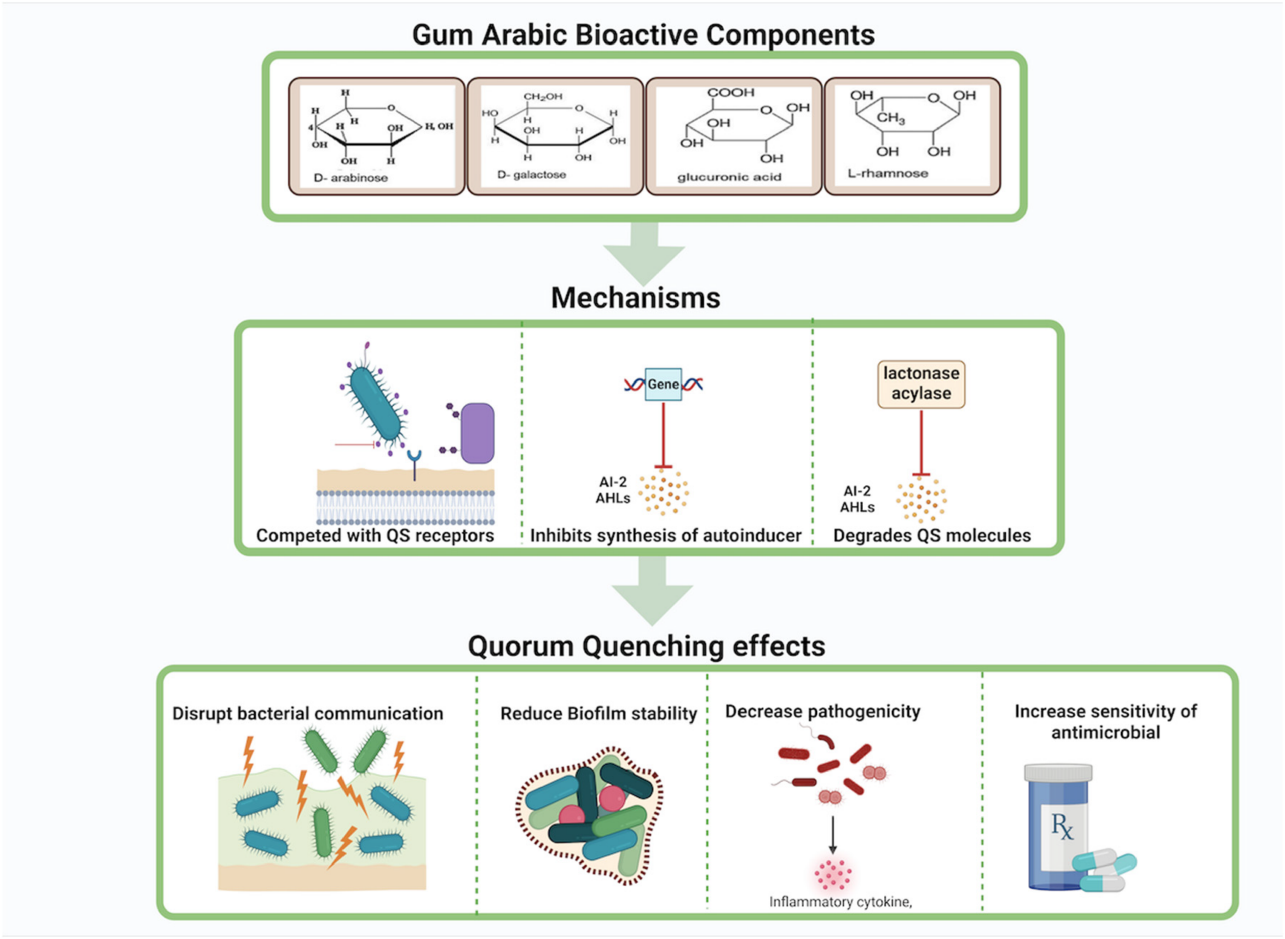
Gum Arabic's bioactive components exhibit quorum quenching effects by competing with quorum sensing receptors, inhibiting the synthesis of autoinducers, and degrading quorum sensing molecules. These mechanisms disrupt bacterial communication, reduce biofilm stability, decrease pathogenicity, and increase bacterial sensitivity to antimicrobials.Created with BioRender.com.

Arabinogalactan may hinder the synthesis of autoinducers such as N-acyl homoserine lactones (AHLs) in Gram-negative bacteria and autoinducer-2 (AI-2) in both Gram-negative and Gram-positive bacteria. This disruption occurs through the suppression of gene activity responsible for synthesizing these signaling molecules, as well as the degradation of QS signal molecules via lactonases and acylases, thus preventing the accumulation of autoinducers necessary for effective QS (73, 74) (Figure 5).

Galactose, another component of gum Arabic, can modulate pathways involved in the synthesis of QS signal molecules. It may downregulate the expression of genes involved in AHL biosynthesis, leading to reduced production of AHLs and impaired bacterial communication (75) Additionally, galactose can facilitate the production or activation of enzymes that degrade AHLs, further disrupting QS signals and reducing biofilm formation and virulence (76) (Figure 5).

**Figure 6:**
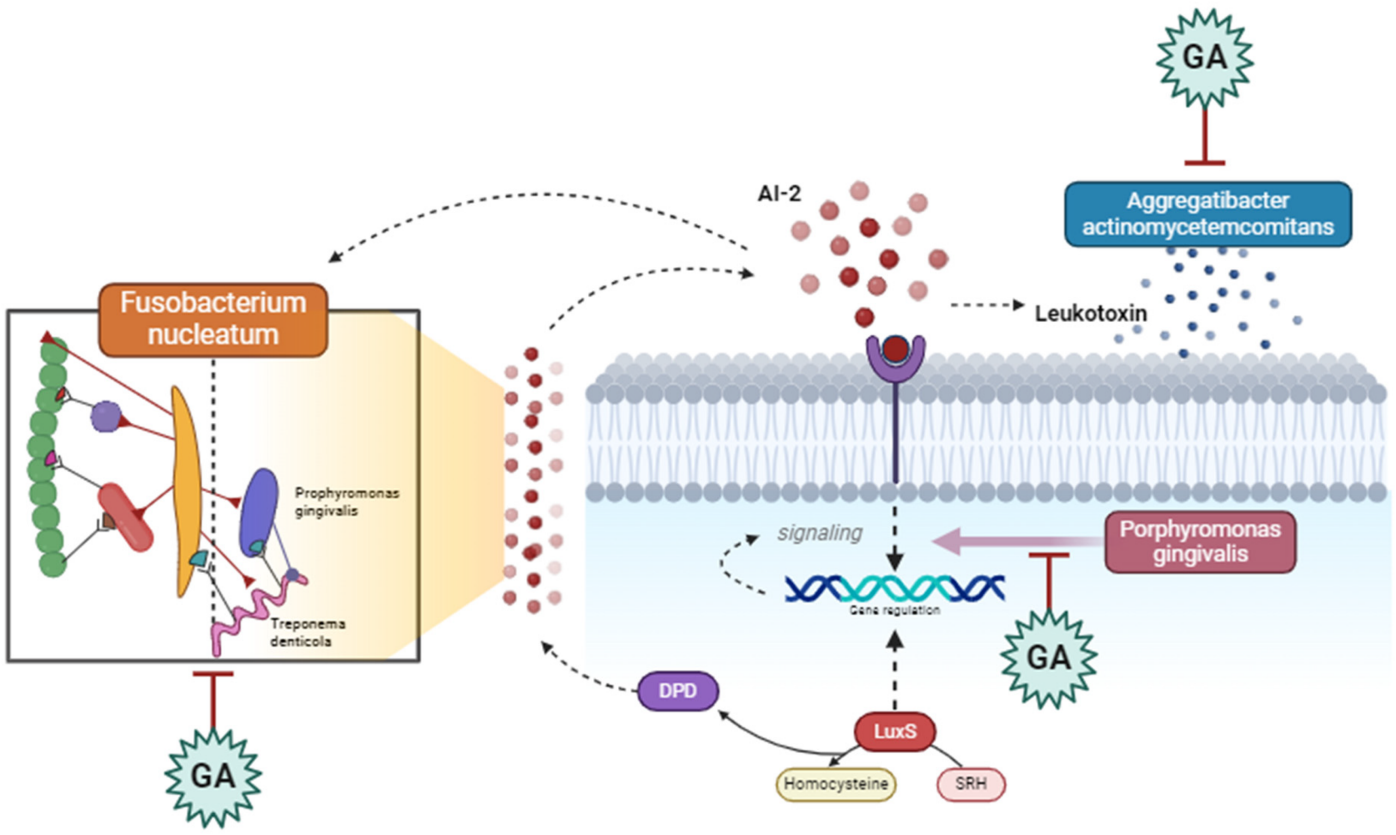
Gum Arabic interferes with the communication between periodontopathogenic bacteria, such as *Fusobacterium nucleatum* and *Porphyromonas gingivalis*, by inhibiting quorum sensing signals and leukotoxin production. It also modulates the activity of *Aggregatibacter actinomycetemcomitans*, reducing virulence factors and downregulating genes involved in pathogenicity, ultimately disrupting bacterial interactions and reducing pathogenic effects in periodontal disease. Created with BioRender.com.

Glycoproteins in gum Arabic, composed of protein backbones bound to carbohydrate chains, can also interfere with QS pathways by inhibiting the expression of genes involved in autoinducer biosynthesis (77). These glycoproteins may either have enzymatic activity or support the synthesis of enzymes that hydrolyze AHLs, preventing the buildup of functional QS signals (78). Moreover, glycoproteins might compete for QS receptors, blocking autoinducers from binding and activating these receptors, thereby suppressing QS-regulated gene expression and reducing biofilm stability and pathogenicity (79, 80) (Figures 5, 6).

## Plausible effects of Gum Arabic on P. gingivalis, A. actinomycetemcomitans, and T. forsythia

Gum Arabic has plausible effects on key periodontal pathogens such as *Porphyromonas gingivalis* (P. gingivalis), *Aggregatibacter actinomycetemcomitans* (A. actinomycetemcomitans), and *Tannerella forsythia* (T. forsythia) (81, 82). These bacteria are significant contributors to periodontal diseases, such as gingivitis and periodontitis, due to their ability to form biofilms and utilize quorum sensing (QS) to coordinate virulence factors. GA's properties suggest it could be a valuable adjunct in managing periodontal diseases by targeting these pathogens (83). According to the research, (GA) has various positive effects on periodontal infections. GA suppresses Porphyromonas gingivalis’ generation of gingipains, which are proteolytic enzymes that contribute to tissue damage and immune evasion. It also interferes with *P. gingivalis’* initial adherence to surfaces and disturbs existing biofilms, making them more susceptible to antimicrobial treatments (84). Furthermore, GA's anti-inflammatory qualities aid in minimizing inflammation produced by *P. gingivalis* infections, minimizing tissue damage, and promoting healing. GA components like D-galactose impede AI-2-mediated quorum sensing, limiting the pathogen's capacity to build biofilms and create virulence factors (81). GA also has direct antibacterial action against A. actinomycetemcomitans, reducing its viability and colonization capacity and disrupting the biofilm extracellular matrix, hence increasing the efficiency of mechanical debridement and antimicrobial treatments (84). Furthermore, GA's anti-inflammatory and antibacterial activities assist in diminishing the overall pathogenicity of *T. forsythia*, providing a better oral environment. *F. nucleatum* plays a central role in the formation and maturation of dental biofilms, acting as a bridge organism that facilitates the integration of early and late colonizers in the biofilm community. GA's properties suggest it could be a valuable adjunct in managing periodontal diseases by targeting *F. nucleatum*. GA can disrupt quorum sensing (QS), the communication mechanism used by *F. nucleatum* to regulate gene expression related to virulence and biofilm formation (63).

**Figure 7:**
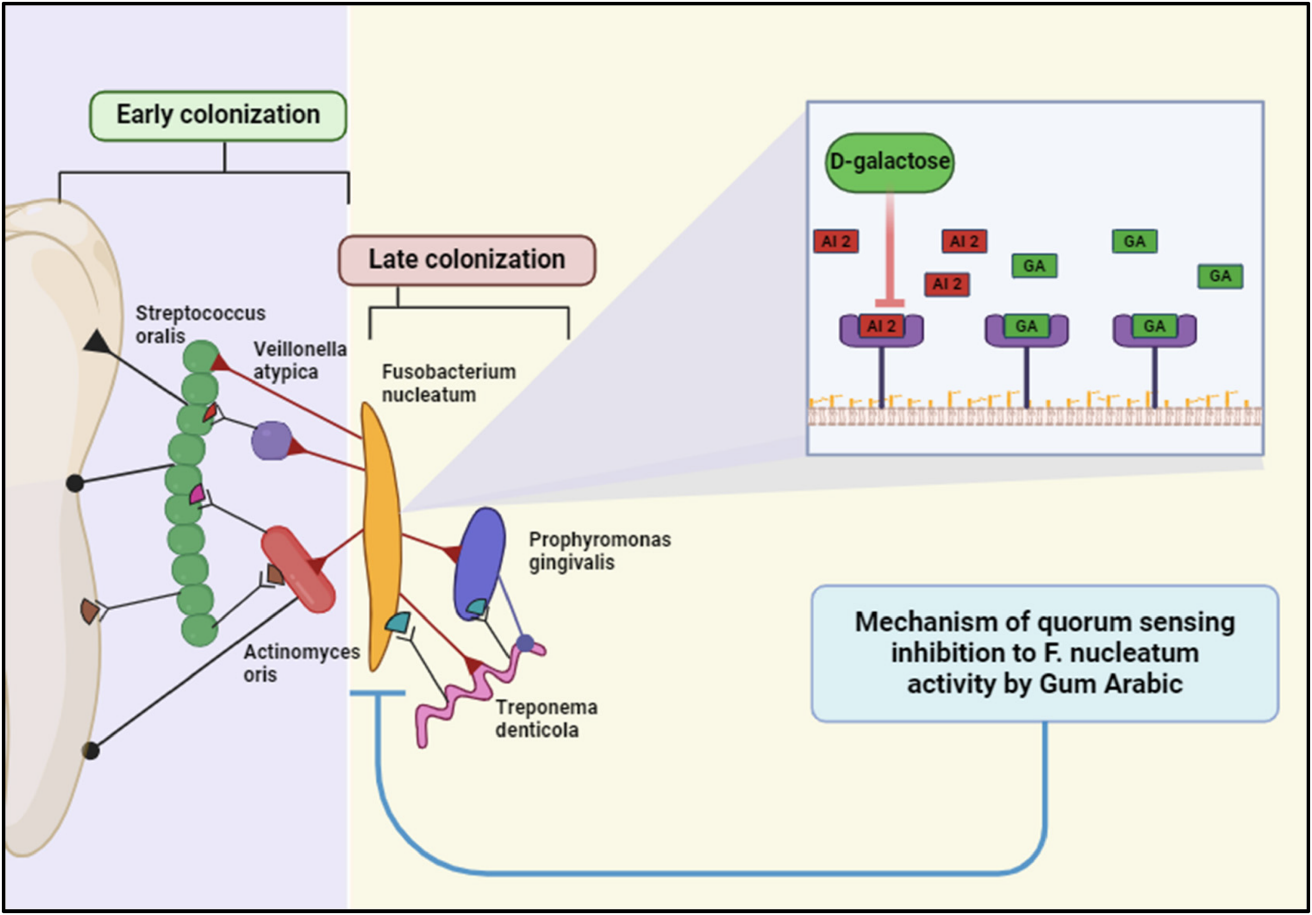
Gum Arabic inhibits *Fusobacterium nucleatum* quorum sensing via its D-galactose component, disrupting bacterial communication and biofilm maturation. This interference impairs the colonization and pathogenicity of periodontal bacteria, including late colonizers like *Porphyromonas gingivalis*. Created with BioRender.com.

Components of GA, such as D-galactose, interfere with the production of autoinducers like AI-2. This disruption prevents *F. nucleatum* from reaching the critical concentration needed to trigger QS-regulated activities (81). (Figure 7). Considering this GA can also block the receptors that detect these autoinducers, effectively silencing the QS signals and preventing the coordination necessary for biofilm maturation and virulence factor production. Furthermore, GA claims to reduce the ability of *F. nucleatum* to adhere to tooth surfaces and other bacteria within the biofilm. This is a crucial step in preventing biofilm formation and maturation (28).

## Evidence supporting Gum Arabic’s effects on periodontal health

GA has demonstrated promising effects on periodontal health, as evidenced by multiple clinical studies. 2-blind crossover trials evaluated the antiplaque properties of GA-containing chewing gum, finding that participants using GA gum exhibited a significant reduction in plaque accumulation compared to the control group (85). Additionally, a randomized controlled trial assessed the efficacy of GA as an adjunct to scaling and root planing (SRP) in treating chronic periodontitis. This study revealed notable improvements in clinical parameters such as reduced gingival inflammation and bleeding in the GA group compared to SRP alone (86). Similarly, another randomized clinical trial investigated the clinical and microbiological benefits of commercially available GA gel and powder in managing gingivitis. Participants using GA products showed significant reductions in gingival index scores and overall inflammation compared to the placebo group (17). These findings underscore GA’s potential as a valuable adjunctive therapy in periodontal treatment, offering a natural and effective means to enhance oral health outcomes.

### Therapeutic applications of Gum Arabic

**Figure 8:**
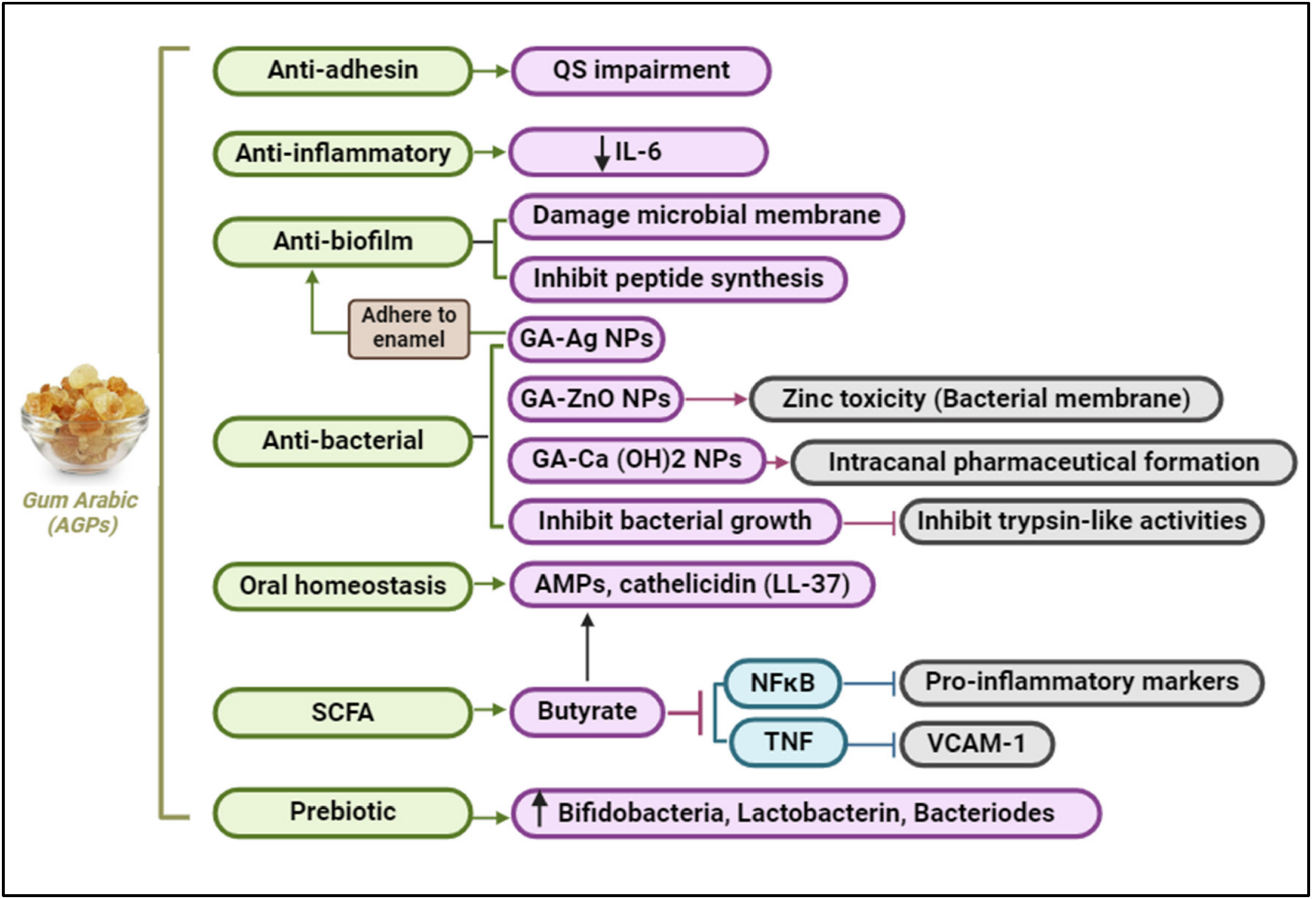
Gum Arabic supports oral health by impairing quorum sensing, inhibiting biofilm formation, reducing inflammation, and exerting antibacterial effects. It also promotes oral homeostasis and prebiotic activity, fostering beneficial bacteria and reducing pro-inflammatory markers. Created with BioRender.com.

• **Preventive and Adjunctive Therapy**: GA can be integrated into dental care products, such as mouthwashes, gels, or slow-release formulations, to enhance plaque control and reduce gingival inflammation. Combining GA with conventional periodontal therapies may offer a synergistic approach to managing periodontal diseases (18, 87).
• **Nanotechnology and Gum Arabic**: Recent studies have explored the use of Gum Arabic (GA) in nanotechnology, particularly in the development of GA-ZnO (Zinc Oxide) nanoparticles, which exhibit enhanced antibacterial properties (88). This integration of GA into nanotechnology has shown promising results in the context of periodontal health. GA-ZnO nanoparticles have demonstrated significant antibacterial activity against a range of periodontal pathogens, including *Porphyromonas gingivalis*, *Aggregatibacter actinomycetemcomitans*, and *Tannerella forsythia*. The zinc oxide component disrupts bacterial cell membranes, leading to cell lysis and death. The combination of GA and ZnO enhances the overall antibacterial effect. GA acts as a stabilizer and dispersing agent, increasing the effectiveness of ZnO nanoparticles. GA-ZnO nanoparticles can also prevent the initial adhesion of bacteria to surfaces, a critical step in biofilm formation. This property is particularly beneficial in maintaining oral hygiene and preventing periodontal diseases. These nanoparticles can penetrate existing biofilms, disrupting their structure and making the bacteria more susceptible to antimicrobial agents and mechanical removal. GA-ZnO nanoparticles can reduce the levels of pro-inflammatory cytokines such as IL-6 and TNF-α (89, 90) (Figure 8). This helps to mitigate the inflammatory response associated with periodontal diseases. GA-ZnO nanoparticles create a less conducive environment for periodontal pathogens by modulating the host immune response, thereby reducing their virulence (91, 92).

## Limitations & future directions

Studies have demonstrated that GA can reduce plaque accumulation and improve gingival inflammation indexes. For example, GA has been found to inhibit the adhesion of bacteria to tooth surfaces, thereby reducing plaque formation (86–88). Additionally, GA exhibits anti-inflammatory properties, leading to improved gingival health and reduced bleeding on probing (87).

However, the evidence supporting GA's effectiveness in treating periodontitis remains limited Most studies have focused on its role in plaque control and gingivitis, with fewer (86) investigations exploring its impact on the more severe forms of periodontal disease.

Recent studies have provided empirical evidence supporting the QS inhibitory effects of polysaccharides and polyphenols. For instance, a study by Singh et al. demonstrated that polysaccharides from other natural sources could bind to and neutralize AHLs, effectively disrupting QS and reducing biofilm formation in Escherichia coli (93).

Another study by Mazzantini et al. highlighted the anti-QS activity of polyphenols in Staphylococcus aureus, leading to decreased biofilm formation and virulence (94).

To better substantiate the influence of Gum Arabic in modulating quorum sensing and its potential as a therapeutic agent for periodontal disease, future research ought to focus on the following areas:

○ *in vitro* studies Using periodontal pathogens to evaluate GA's effects on biofilms’ development and the generation of quorum-sensing molecules.
○ Animal models to assess the therapeutic effectiveness of Gum Arabic in treating periodontal disease.
○ Examining the combined impact of GA in conjunction with other periodontal treatments to improve overall treatment outcomes.

## Conclusion

While preliminary findings are promising, the role of GA in the treatment of periodontal disease, including periodontitis, requires more robust evidence. Combining GA with conventional periodontal therapies may offer a synergistic approach, but further research is crucial to confirm its effectiveness and define its place in periodontal care.

## What is known about this topic

• Gum Arabic has been investigated as a natural bioactive compound in the process of quorum quenching, which might potentially provide a novel method for controlling periodontal diseases by interrupting bacterial communication. This disturbance may impede the ability of harmful oral biofilms to cause disease without developing antibiotic resistance.
• Quorum quenching may use natural substances such as phytochemicals to inhibit the communication process among bacteria, thereby reducing the likelihood of illness reoccurrence and improving the effectiveness of mechanical therapy.
• The investigation of quorum sensing and its suppression indicates potential uses in dentistry, particularly given the significant worldwide prevalence of oral conditions such as periodontitis.

## What this study adds

• Gum Arabic, has the potential to provide a new approach to treating periodontal disorders by inhibiting quorum sensing.
• Gum Arabic can interfere with the bacterial communication mechanisms that cause the formation and maintenance of harmful biofilms in the mouth.
• This strategy prioritizes the inhibition of virulence factor production in bacterial populations as a crucial aspect of periodontal disease management while avoiding any contribution to the escalating issue of antimicrobial resistance.
